# PMS5: an efficient exact algorithm for the (ℓ, *d*)-motif finding problem

**DOI:** 10.1186/1471-2105-12-410

**Published:** 2011-10-24

**Authors:** Hieu Dinh, Sanguthevar Rajasekaran, Vamsi K Kundeti

**Affiliations:** 1 Department of CSE, University of Connecticut, Storrs, CT 06269, USA

## Abstract

**Background:**

Motifs are patterns found in biological sequences that are vital for understanding gene function, human disease, drug design, etc. They are helpful in finding transcriptional regulatory elements, transcription factor binding sites, and so on. As a result, the problem of identifying motifs is very crucial in biology.

**Results:**

Many facets of the motif search problem have been identified in the literature. One of them is (ℓ, *d*)*-motif search *(or *Planted Motif Search (PMS))*. The PMS problem has been well investigated and shown to be NP-hard. Any algorithm for PMS that always finds all the (ℓ, *d*)-motifs on a given input set is called an *exact *algorithm. In this paper we focus on exact algorithms only. All the known exact algorithms for PMS take exponential time in some of the underlying parameters in the worst case scenario. But it does not mean that we cannot design exact algorithms for solving practical instances within a reasonable amount of time. In this paper, we propose a fast algorithm that can solve the well-known challenging instances of PMS: (21, 8) and (23, 9). No prior exact algorithm could solve these instances. In particular, our proposed algorithm takes about 10 hours on the challenging instance (21, 8) and about 54 hours on the challenging instance (23, 9). The algorithm has been run on a single 2.4GHz PC with 3GB RAM. The implementation of PMS5 is freely available on the web at http://www.pms.engr.uconn.edu/downloads/PMS5.zip.

**Conclusions:**

We present an efficient algorithm PMS5 that uses some novel ideas and combines them with well-known algorithm PMS1 and PMSPrune. PMS5 can tackle the large challenging instances (21, 8) and (23, 9). Therefore, we hope that PMS5 will help biologists discover longer motifs in the futures.

## 1 Background

The discovery of patterns in DNA, RNA, and protein sequences has led to the solution of many vital biological problems. For instance, the identification of patterns in nucleic acid sequences has resulted in the determination of open reading frames, identification of promoter elements of genes, identification of intron/exon splicing sites, identification of SH RNAs, location of RNA degradation signals, identification of alternative splicing sites, etc. In protein sequences, patterns have proven to be extremely helpful in domain identification, location of protease cleavage sites, identification of signal peptides, protein interactions, determination of protein degradation elements, identification of protein trafficking elements, discovery of short functional motifs, etc. Motifs are patterns found in biological sequences that are vital for understanding many biological subjects like gene function, human disease, drug design etc. As a result, the identification of motifs plays an important role in biological studies. The motif search problem has been attracting many researchers. In the literature, many versions of the motif search problem have been enumerated. Examples include *Simple Motif Search (SMS), Planted Motif Search (PMS) - also known as *(ℓ, *d*)*-motif search*, and *Edit-distance-based Motif Search (EMS) *(see e.g., [1]). In this paper, we will focus on the PMS problem (or PMS for short).

### The definition of Planted Motif Search (PMS)

PMS is stated as follows. It takes as input *n *sequences, two integers ℓ and *d*. For simplicity, we assume that the length of each sequence is *m*. The problem is to identify all strings *M *of length ℓ such that *M *occurs in each of the *n *sequences with at most *d *mismatches. Formally, string *M *has to satisfy the following constraint: there exists a string *M*_*i *_of length *l *in sequence *i*, for every *i *(1 ≤ *i *≤ *n*), such that the number of mismatches between *M *and *M*_*i *_is less than or equal to *d*. The number of mismatches between two strings of equal length is known as the *Hamming distance *between them. *String M is called a motif*. For example, if the input sequences are GCGCGAT, CAGGTGA, and CGATGCC; ℓ = 3; and *d *= 1, then GAT and GTG are some of the (*l, d*)-motifs. PMS is a well-studied problem and it has been shown to be NP-hard. As a result, all known exact algorithms for PMS take exponential time in some of the underlying parameters in the worst case. Two kinds of algorithms have been proposed in the literature for PMS: exact and *approximate*. While an exact algorithm always finds all the motifs, an approximate algorithm may not always find all the motifs. Typically, approximate algorithms tend to be faster than exact algorithms. Some example approximate algorithms are due to Bailey and Elkan [2], Buhler and Tompa [3], Lawrence et al. [4], Pevzner and Sze [5], and Rocke and Tompa [6]. These algorithms are based on local search techniques such as Gibbs sampling, expectation optimization, etc. The WINNOWER algorithm of [5] is based on finding cliques in a graph. Some other approximate algorithms are: PROJECTION [3], MULTIPROFILER [7], PatternBranching [8], CONSENSUS [9], GibbsDNA [4], MEME [2], and ProfileBranching [8].

Although approximate algorithms are acceptable in some cases in practice, exact algorithms are preferable since they are guaranteed to report all the (*l, d*)-motifs. For biologists, the motifs found by an algorithm could be much more important than its run time. As a result, we focus in this paper on efficient exact algorithms. Some exact algorithms known for PMS are: [10-18], and [19].

Buhler and Tompa [3] have employed PMS algorithms to find known transcriptional regulatory elements upstream of several eukaryotic genes. In particular, they have used orthologous sequences from different organisms upstream of four different genes: preproinsulin, dihydrofolate reductase (DHFR), metallothioneins, and c-fos. These sequences are known to contain binding sites for specific transcription factors. The authors point out the differences between experimental data and synthetic data that PMS algorithms are typically tested with. For example, the background DNA in experimental data is not random. Their algorithm successfully identified the experimentally determined transcription factor binding sites. They have used the values of l = 20 and d = 2. The same sites have also been found using our PMS2 algorithm [11]. The algorithm of [3] is an approximation algorithm whereas PMS2 is an exact algorithm. Buhler and Tompa have also employed their algorithm to solve the ribosome binding site problem for various prokaryotes [3]. This problem is even more challenging since here the number of input sequences could be in the thousands.

Eskin and Pevzner [13] used PMS algorithms to find composite regulatory patterns. They point out that traditional pattern finding techniques (on unaligned DNA sequences) concentrate on identifying high-scoring monads. A regulatory pattern could indeed be a combination of multiple and possibly weak monads. They employ MITRA (a PMS algorithm) to locate regulatory patterns of this kind. The algorithm is demonstrated to perform well on both synthetic and experimental data sets. For example, they have employed the upstream regions involved in purine metabolism from three Pyrococcus genomes. They have also tested their algorithm on four sets of S.cerevisiae genes which are regulated by two transcription factors such that the transcription factor binding sites occur near each other. Price and Pevzner [8] have employed their PatternBranching PMS technique on a sample containing CRP binding sites in E.coli, upstream regions of many organisms of the eukaryotic genes: preproinsulin, DHFR, metallothionein, & c-fos, and a sample of promoter regions from yeast. They report finding numerous motifs in these sequences.

The performance of an exact algorithm is typically evaluated on random benchmark data generated as follows. Twenty input DNA sequences, each of length 600, are generated randomly from the alphabet Σ = {*A, C, G, T*}. A motif *M *of length ℓ is also generated randomly and planted in each of the input sequences within a Hamming distance of *d *to make sure that there always exists a motif in the input. Based on the values of ℓ and *d*, certain instances of PMS have been identified to be *challenging*. An instance is challenging if the expected number of the motifs that occur by random chance (in addition to the planted one) is one or more. For example, the following instances are challenging: (9, 2), (11, 3), (13, 4), (15, 5), (17, 6), (19, 7), (21,8), (23, 9), etc.

To compare the performance of exact algorithms, the challenging instances are commonly used. For example, the exact algorithm MITRA of [8] can solve the challenging instances (9, 2), (11, 3), and (13, 4). It takes either too much time or too much memory on the challenging instance (15, 5) or any larger instances. Both the exact algorithm Voting in [20] and the exact algorithm RISOTTO in [21] can solve the challenging instances up to (15, 5). In most of the cases, Voting runs faster than RISOTTO. The best up-to-date exact algorithm is Pampa given in [10]. Pampa can solve the challenging instance (19, 7) within about 4.8 hours. The second best exact algorithm is PMSPrune [22] that can solve the challenging instance (19, 7) within about 10 hours.

In this paper we present an exact algorithm (named PMS5) that can solve the challenging instances (21, 8) and (23, 9). PMS5 takes about 10 hours on the challenging instance (21, 8) and about 54 hours on the challenging instance (23, 9). These run times are on a single 2.4GHz PC with 3GB of RAM. To the best of our knowledge, no other exact algorithm can solve these instances.

## 2 Methods

### 2.1 Notations and Definitions

In this section we introduce some notations and definitions that will help us describe our algorithm clearly.

**Definition 2.1**. *A string x *= *x*[1] ... *x*[ℓ] *of length *ℓ *is called an *ℓ-*mer*.

**Definition 2.2**. *Given two strings x and y of equal length, we say the Hamming distance between x and y, denoted by d*_*H*_(*x, y*). *is the number of mismatches between them*,

**Definition 2.3**. *Given a string x *= *x*[1] ... *x*[ℓ], *we define the **d-neighborhood **of x, B*_*d*_(*x*), *to be *{*y *| *d*_*H*_(*x, y*) ≤ *d*}.

Note that $|B_{d}(x)|=\sum_{i=0}^{d}\left({}_{i}^{\ell }\right){\left(|\Sigma |-1\right)}^{i}$, where Σ is the alphabet of interest. Notice that *B*_*d*_(*x*) depends only on ℓ, *d *and |Σ|. For this reason, we define $N(\ell ,d)=\sum_{i=0}^{d}\left({}_{i}^{\ell }\right){\left(|\Sigma |-1\right)}^{i}$.

**Definition 2.4**. *Given two strings x *= *x*[1] ... *x*[ℓ] *and s *= *s *[1] ... *s*[*m*] *with *ℓ <*m:*

1. *We use the notation x *∈_ℓ _*s if x is a substring of s (equivalently, s *= *αxβ for some strings α and β). We also say that x is an *ℓ-*mer of s*.

2. *We define *${\bar{d}}_{H}\left(x,s\right)=\min_{r\in_{\ell }s}d_{H}\left(x,r\right)$.

**Definition 2.5**. *Given a string x = x*[1] ... *x*[ℓ] *and a set of strings *$S=\left\{s_{1},...,s_{n}\right\}$*with *|*s*_*i*_| = *m for i *= 1, ..., *n and *ℓ <*m, we define *${\bar{d}}_{H}\left(x,S\right)=\underset{1\leq i\leq n}{\max}{\bar{d}}_{H}\left(x,s_{i}\right)$.

It is easy to see that *x *is an (ℓ, *d*)-motif of S if and only if ${\bar{d}}_{H}\left(x,S\right)\leq d$.

**Definition 2.6**. *Given a set of strings *$S=\left\{s_{1},...,s_{n}\right\}$, *we define *$M_{\ell ,d}\left(S\right)$*to be the set of *(*l, d*) *motifs of *S.

The goal of PMS is to compute $M_{\ell ,d}\left(S\right)$, given ℓ, *d *and S.

### 2.2 PMS5 - A fast algorithm

The idea of our algorithm is based on the following observations about $M_{\ell ,d}\left(S\right)$.

**Observation **2.1. *Let *S, $S^{\prime }$*and *$S^{\prime \prime }$*be three sets of strings such that *$S=S^{\prime }\cup S^{\prime \prime }$*and *$S^{\prime }\cap S^{\prime \prime }=\emptyset$. *It is easy to observe that *$M_{\ell ,d}\left(S\right)=M_{\ell ,d}\left(S^{\prime }\right)\cap M_{\ell ,d}\left(S^{\prime \prime }\right)$.

**Observation **2.2. *For any string s*, $M_{\ell ,d}\left(\left\{s\right\}\right)={⋃}_{x\in_{\ell }s}B_{d}\left(x\right)$.

From Observation 2.1 and Observation 2.2, we can obtain the following observation.

**Observation **2.3. *Let *$S^{*}=S\backslash \left\{s_{1}\right\}=\left\{s_{2},...,s_{n}\right\}$. *We have *$M_{\ell ,d}\left(S\right)={⋃}_{x\in_{\ell }s_{1}}\left[B_{d}\left(x\right)\cap M_{\ell ,d}\left(S^{*}\right)\right]$.

Observation 2.3 tells us that $M_{\ell ,d}\left(S\right)$ can be computed from $B_{d}\left(x\right)\cap M_{\ell ,d}\left(S^{*}\right)$.

Without loss of generality, we can assume that the size of $S^{*}$ is even, i.e., $|S^{*}|=n-1=2p$, for some integer *p*. Otherwise we can add a copy of *s*_*n *_into $S^{*}$, in which case $M_{\ell ,d}\left(S^{*}\right)$ will remain the same. Next, we partition $S^{*}$ into pairs of strings $S_{1},...,S_{p}$, where $S_{k}=\left\{s_{2k},s_{2k+1}\right\}$ for *k *= 1 ... *p*. From Observations 2.1 and 2.2, we can make the following observations.

Observation 2.4.

$$B_{d}\left(x\right)\cap M_{\ell ,d}\left(S^{*}\right)={⋂}_{1\leq k\leq p}\left[B_{d}\left(x\right)\cap M_{\ell ,d}\left(S_{k}\right)\right].$$

Observation 2.5.

$$B_{d}\left(x\right)\cap M_{\ell ,d}\left(S_{k}\right)={⋃}_{y\in_{\ell }{s_{2}}_{k},z\in_{\ell }s_{2k+1}}\left[B_{d}\left(x\right)\cap B_{d}\left(y\right)\cap B_{d}\left(z\right)\right].$$

Based on the above observations, we note that the process of computing $M_{\ell ,d}\left(S\right)$ can be reduced to computing *B*_*d*_(*x, y, z*) = *B*_*d*_(*x*) ∩ *B*_*d*_(*y*) ∩ *B*_*d*_(*z*) repeatedly. We will discuss how to compute *B*_*d*_(*x, y, z*) efficiently in Section 2.2.2. The pseudocode of our algorithm PMS5 is given below.

                                                          **Algorithm **PMS5

1:  **for **each *x *∈_ℓ _*s*_1 _**do**

2:     **for ***k *= 1 to $p=\left\lfloor \frac{n-1}{2}\right\rfloor$**do**

3:      Q←∅.

4:      **for **each *y *∈_ℓ _*s*_2*k *_and *z *∈_ℓ _*s*_2*k*+1 _**do**

5:         Compute *B*_*d*_(*x, y, z*) = *B*_*d*_(*x*) ∩ *B*_*d*_(*y*) ∩ *B*_*d*_(*z*).

6:         *Q *← *Q *∪ *B*_*d*_(*x, y, z*).

7:      **end for**

8:      **if ***k *= 1 **then**

9:       *Q*' ← *Q*.

10:      **else**

11:        *Q*' ← *Q*' ∩ *Q*.

12:      **end if**

13:       **if **|*Q*'| is small enough **then**

14:         **break **the for loop.

15:       **end if**

16:  **end for**

17:  **for **each *r *∈ *Q*' **do**

18:      **if **${\bar{d}}_{H}\left(r,S\right)\leq d$**then**

19:        Output *r *as an (ℓ, *d*) motif.

20:      **end if**

21:    **end for**

22:  **end for**

In the pseudo code, the process of computing $B_{d}\left(x\right)\cap M_{\ell ,d}\left(S_{k}\right)$ for each *k *is from line 3 to line 7. After line 7, *Q *is actually $B_{d}\left(x\right)\cap M_{\ell ,d}\left(S_{k}\right)$. Within the loop at line 2, *Q*' is $B_{d}\left(x\right)\cap M_{\ell ,d}\left(S_{1}\right)\cap \cdots \cap M_{\ell ,d}\left(S_{k}\right)$ for each *k *after line 12. At line 13, if |*Q*'| is less than a certain threshold, the algorithm simply exits the loop and will not try other values of *k*. In practice, we set the threshold to be between 5000 and 10000. From line 17 to line 21, the algorithm checks if each string *r ***∈ ***Q*' is actually an (ℓ, *d*)-motif or not. To check if ${\bar{d}}_{H}\left(r,S\right)\leq d$ for any *r*, we only have to use the remaining sequences (*s*_2*k*+2_, *s*_2*k*+3_, ..., *s*_*n*_).

#### 2.2.1 Analysis

##### PMS5 is correct

From the observations, it is straightforward to see that PMS5 outputs $M_{\ell ,d}\left(S\right)$. Therefore, PMS5 is correct.

##### The worst-case run time of PMS5

**Theorem 2.1**. *The worst-case run time of PMS5 is *$O\left(nm^{3}dN\left(\ell ,d\right)\right)$. *Recall that *$N(\ell ,d)=|B_{d}(x)|=\sum_{i=0}^{d}\left({}_{i}^{\ell }\right){\left(|\Sigma |-1\right)}^{i}$.

*Proof*. It is easy to see that the run time of PMS5 is dominated by the computation time of *B*_*d*_(*x, y, z*) in line 5. In Section 2.2.2, we will show that *B*_*d*_(*x, y, z*) can be computed in *O*(ℓ + *d*|*B*_*d*_(*x, y, z*)|) time. In the extreme case in which $x=y=z,|B_{d}(x,y,z))|=|B_{d}(x)|=N(\ell ,d)$. Since N(ℓ,d) is much larger than ℓ, the computation time of *B*_*d*_(*x, y, z*) is O(dN(ℓ,d)). Also, it is straightforward to see that the number of times *B*_*d*_(*x, y, z*) is computed is at most $\frac{n}{2}{\left(m-\ell +1\right)}^{3}$. Hence, the run time of PMS5 is $O\left(nm^{3}dN\left(\ell ,d\right)\right)$.

##### The expected run time of PMS5

We can compute the expected run time of of PMS5 by computing the expected value of *B*_*d*_(*x, y, z*). Let *x, y*, and *z *be three random ℓ-mers. How many ℓ-mers are there that are at a distance of ≤ *d *from each of *x, y*, and *z*? Let *u *be a random ℓ-mer. Prob.$[d_{H}(x,u)\leq d]=p_{\ell ,d}=\sum_{i=0}^{d}\left({}_{i}^{\ell }\right){\left(3/4\right)}^{i}{\left(1/4\right)}^{\ell -i}$. This means that Prob.$\left[d_{H}\left(x,u\right)\leq d\&d_{H}\left(y,u\right)\leq d\&d_{H}\left(z,u\right)\leq d\right]=p_{l,d}^{3}$. Therefore, the expected number of *u*'s such that *u *is at a distance of ≤ *d *from each of *x, y*, and *z, E*[*B*_*d*_(*x, y, z*)], is $4^{\ell }p_{\ell ,d}^{3}$.

As a result, the expected run time of PMS5 is $O\left(nm^{3}d4^{\ell }p_{\ell ,d}^{3}\right)$, where $p_{\ell ,d}=\sum_{i=0}^{d}\left({}_{i}^{\ell }\right){\left(3/4\right)}^{i}{\left(1/4\right)}^{\ell -i}$.

Table 1 gives a comparison between N(ℓ,d) and *E*[*B*_*d*_(*x, y, z*)] for different values of ℓ and *d*.

**Table 1:** A comparison between N(ℓ,d) and *E*[*B*_*d*_(*x, y, z*)] for different values of ℓ and *d*

ℓ	*d*	N(ℓ,d)	*E*[*B*_*d*_(*x, y, z*)]
9	2	352	6.35 × 10^-4^

11	3	4,984	7.04 × 10^-3^

13	4	66,379	6.49 × 10^-2^

15	5	853,570	5.39 × 10^-1^

17	6	1.07 × 10^7^	4.20

19	7	1.33 × 10^8^	3.12 × 10

21	8	1.63 × 10^9^	2.26 × 10^2^

23	9	1.99 × 10^10^	1.60 × 10^3^

#### 2.2.2 Computing the intersection of the d-neighborhoods

In this section, we consider the problem of computing the intersection of the *d*-neighborhoods *B*_*d*_(*x, y, z*). Given three ℓ-mers *x, y, z *and integer number *d*, we would like to list all of the ℓ-mers in *B*_*d*_(*x, y, z*). In this section we offer an algorithm FULLPRUNE for this task that runs in *O*(ℓ + *d*|*B*_*d*_(*x, y, z*)|) time.

FULLPRUNE is the heart of algorithm PMS5. The idea of FULLPRUNE is as follows. We first represent *B*_*d*_(*x*) as a tree $T_{d}\left(x\right)$ in which each node is an ℓ-mer in *B*_*d*_(*x*) and its root is the ℓ-mer *x*. The depth of $T_{d}\left(x\right)$ is *d*. We will describe $T_{d}\left(x\right)$ in detail later. We traverse $T_{d}\left(x\right)$ in a depth-first manner. At each node *t *during the traversal, we output *t *if *t *is in *B*_*d*_(*y*) ∩ *B*_*d*_(*z*). We also check if there is a descendent *t*' of *t *such that *t*' is in *B*_*d*_(*y*) ∩ *B*_*d*_(*z*). If there is no such descendent, we prune the subtree rooted at node *t*. We will show that checking the existence of such a descendent can be done quickly in *O*(1) time, later. Formally, $T_{d}\left(x\right)$ is constructed from the following rules.

**Rules **to construct $T_{d}\left(x\right)$.

1. Each node in $T_{d}\left(x\right)$ is a pair (*t, p*) where *t *= *t*[1] ... *t*[ℓ] is an ℓ-mer and *p *is an integer between 0 and ℓ such that *t*[*p *+ 1] ... *t*[ℓ] = *x*[*p *+ 1] ... *x*[ℓ]. We refer to a node (*t, p*) as ℓ-mer *t *if *p *is clear.

2. Let *t *= *t*[1] ... *t*[ℓ] and *t*' = *t*'[1] ... *t*'[ℓ]. A node (*t, p*) is the parent of a node (*t*', *p*') if and only if

(a) *p*' >*p*.

(b) *t*'[*p*'] ≠ *t*[*p*'] (From Rule 1, *t*[*p*'] = *x*[*p*']).

(c) *t*'[*i*] = *t*[*i*] for any *i *≠ *p*'

3. The root of $T_{d}\left(x\right)$ is (*x*, 0).

4. The depth of $T_{d}\left(x\right)$ is *d*.

**Figure 1:**
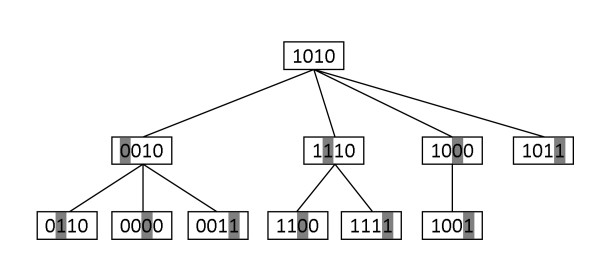
**$T_{2}\left(1010\right)$ with alphabet Σ = {0,1}**. The value of *p *at each node is the location of its shaded letter. For example, *p *= 2 at node 1110, *p *= 3 at node 0000.

Clearly, each ℓ-mer in *B*_*d*_(*x*) is uniquely associated with a node in $T_{d}\left(x\right)$ and vice versa. Figure 1 illustrates the tree $T_{2}\left(1010\right)$ with alphabet Σ = {0, 1}.

It is not hard to see that $T_{d}\left(x\right)$ has the following properties.

**Properties **of $T_{d}\left(x\right)$.

1. If a node (*t*', *p*') is a child of a node (*t, p*), then *d*_*H*_(*x, t*') - *d*_*H*_(*x, t*) = *d*_*H*_(*t, t*') = 1. As a result, if a node (*t, p*) at level *h*, then *d*_*H*_(*x, t*) = *h*.

2. Consider two nodes (*t, p*) and (*t*', *p*') with *t *= *t*[1] ... *t*[ℓ] and *t*' = *t*'[1] ... *t*'[ℓ]. Then (*t*', *p*') is a descendent of (*t, p*) if and only if:

(a) *p*' >*p*.

(b) *t*'[1] ... *t*'[*p*] = *t*[1] ... *t*[*p*].

(c) *d*_*H*_(*x, t*') ≤ *d*.

Now we consider the subproblem of checking whether there is a descendent (*t*', *p*') of (*t, p*) such that *t*' is in *B*_*d*_(*y*) ∩ *B*_*d*_(*z*). Solving the subproblem is very crucial for FULLPRUNE because it will help us know beforehand for sure which nodes should be explored. The second property above is important to solve the subproblem. Let *t *= *t*[1] ... *t*[ℓ], *x *= *x*[1] ... *x*[ℓ],*y *= *y*[1] ... *y*[ℓ] and *z *= *z*[1] ... *z*[ℓ]. Let *t*_1 _= *t*[1] ... *t*[*p*] and *t*_2 _= *t*[*p *+ 1] ... *t*[ℓ]. We define *x*_1_, *x*_2_, *y*_1_, *y*_2_, *z*_1 _and *z*_2_, similarly. Notice that *x*_2 _= *t*_2_. Because of the second property *t*' must have the form *t*' = *t*_1_*w*, where *w *is an (ℓ - *p*)-mer. Therefore, there is a descendent (*t*', *p*') of (*t, p*) such that *t*' is in *B*_*d*_(*y*) ∩ *B*_*d*_(*z*) if and only if there is an (ℓ - *p*)-mer *w *satisfying the following constraints:

1. *d*_*H*_(*x, t*') = *d*_*H*_(*x*_1_, *t*_1_) + *d*_*H*_(*x*_2_, *w*) ≤ *d*.

2. *d*_*H*_(*y, t*') = *d*_*H*_(*y*_1_, *t*_1_) + *d*_*H*_(*y*_2_, *w*) ≤ *d*.

3. *d*_*H*_(*z, t*') = *d*_*H*_(*z*_1_, *t*_1_) + *d*_*H*_(*z*_2_, *w*) ≤ *d*.

We will show that the constraints can be expressed by an integer linear program of ten variables. Each location *i *in *x*_2_, *y*_2 _and *z*_2 _is one of five types.

• Type 1 (or Type aaa): *x*_2_[*i*] = *y*_2_[*i*] = *z*_2_[*i*].

• Type 2 (or Type aab): *x*_2_[*i*] = *y*_2_[*i*] ≠ *z*_2_[*i*].

• Type 3 (or Type aba): *x*_2_[*i*] = *z*_2_[*i*] ≠ *y*_2_[*i*].

• Type 4 (or Type baa): *x*_2_[*i*] ≠ *y*_2_[*i*] = *z*_2_[*i*].

• Type 5 (or Type abc): *x*_2_[*i*] ≠ *y*_2_[*i*], *x*_2_[*i*] ≠ *z*_2_[*i*], *y*_2_[*i*] ≠ *z*_2_[*i*].

Let *n*_1 _(resp. *n*_2_, *n*_3_, *n*_3_, *n*_4_, and *n*_5_) be the number of locations of Type 1 (resp. Type 2, Type 3, Type 4, and Type 5). Given *x*_2_, *y*_2 _and *z*_2_, *n*_*j *_is determined for *j *= 1 ... 5. Notice that *n*_1 _+ ··· + *n*_5 _= ℓ - *p*.

Consider any (ℓ - *p*)-mer *w *= *w*[1] ... *w*[ℓ - *p*]. We define the following variables.

• Let *N*_1,*a *_be the number of locations *i *of Type 1 such that *w*[*i*] = *x*_2_[*i*]. We should have *N*_1,*a *_≤ *n*_1_.

• Let *N*_2_,_*a *_(resp. *N*_2,*b*_) be the number of locations *i *of Type 2 such that *w*[*i*] = *x*_2_[*i*] (resp. *w*[*i*] = *z*_2_[*i*]). We should have *N*_2,*a *_+ *N*_2,*b *_≤ *n*_2_.

• Let *N*_3,*a *_(resp. *N*_3_,_*b*_) be the number of locations *i *of Type 3 such that *w*[*i*] = *x*_2_[*i*] (resp. *w*[*i*] = *y*_2_[*i*]). We should have *N*_3,*a *_+ *N*_3,*b *_≤ *n*_3_.

• Let *N*_4,*a *_(resp. *N*_4_,_*b*_) be the number of locations *i *of Type 4 such that *w*[*i*] = *y*_2_[*i*] (resp. *w*[*i*] = *x*_2_[*i*]). We should have *N*_4,*a *_+ *N*_4,*b *_≤ *n*_4_.

• Let *N*_5,*a *_(resp. *N*_5_,_*b*_, *N*_5_,_*c*_) be the number of locations *i *of Type 5 such that *w*[*i*] = *x*_2_[*i*] (resp. *w*[*i*] = *y*_2_[*i*], *w*[*i*] = *z*_2_[*i*]). We should have *N*_5,*a *_+ *N*_5,*b *_+ *N*_5,*c *_≤ *n*_4_.

Now it is straightforward to calculate *d*_*H*_(*x*_2_, *w*) through the variables. The number of mismatches between *x*_2 _and *w *caused by the locations of Type 1 (resp. Type 2, Type 3, Type 4, and Type 5) is *n*_1 _- *N*_1,*a*_, (resp. *n*_2 _- *N*_2,*a*_, *n*_3 _- *N*_3,*a*_, *n*_4 _- *N*_4,*b*_, and *n*_5 _- *N*_5,*a*_). Therefore, *d*_*H*_(*x*_2_, *w*) = (*n*_1 _- *N*_1,*a*_) + (*n*_2 _- *N*_2,*a*_) + (*n*_3 _- *N*_3,*a*_) + (*n*_4 _- *N*_4,*b*_) + (*n*_5 _- *N*_5,*a*_). Similarly, *d*_*H*_(*y*_2_, *w*) = (*n*_1 _- *N*_1,*a*_) + (*n*_2 _- *N*_2,*a*_) + (*n*_3 _- *N*_3,*b*_) + (*n*_4 _- *N*_4,*a*_) + (*n*_5 _- *N*_5,*b*_), and *d*_*H*_(*z*_2_, *w*) = (*n*_1 _- *N*_1,*a*_) + (*n*_2 _- *N*_2,*b*_) + (*n*_3 _- *N*_3,*a*_) + (*n*_4 _- *N*_4,*a*_) + (*n*_5 _- *N*_5,*c*_). So the following integer linear program (ILP) expresses the constraints.

Integer Linear Program (ILP).

1. (*n*_1 _- *N*_1,*a*_) + (*n*_2 _- *N*_2,*a*_) + (*n*_3 _- *N*_3,*a*_) + (*n*_4 _- *N*_4,*b*_) + (*n*_5 _- *N*_5,*a*_) ≤ *d *- *d*_*H*_(*x*_1_, *t*_1_).

2. (*n*_1 _- *N*_1_,_*a*_) + (*n*_2 _- *N*_2,*a*_) + (*n*_3 _- *N*_3,*b*_) + (*n*_4 _- *N*_4,*a*_) + (*n*_5 _- *N*_5,*b*_) ≤ *d *- *d*_*H*_(*y*_1_, *t*_1_).

3. (*n*_1 _- *N*_1,*a*_) + (*n*_2 _- *N*_2,*b*_) + (*n*_3 _- *N*_3_,_*a*_) + (*n*_4 _- *N*_4,*a*_) + (*n*_5 _- *N*_5,*c*_) ≤ *d *- *d*_*H*_(*z*_1_, *t*_1_).

4. *N*_1, *a *_≤ *n*_1_.

5. *N*_2, *a *_+ *N*_2, *b *_≤ *n*_2_.

6. *N*_3, *a *_+ *N*_3, *b *_≤ *n*_3_.

7. *N*_4, *a *_+ *N*_4, *b *_≤ *n*_4_.

8. *N*_5, *a *_+ *N*_5, *b *_+ *N*_5_,_*c *_≤ *n*_5_.

9. All of the variables are non-negative integers.

Clearly, there exists one or more *w*'s satisfying the constraints if and only if the integer linear program has a solution. Notice that *n*_1 _+ *n*_2 _+ *n*_3 _+ *n*_4 _+ *n*_5 _= ℓ - *p*. We can rewrite the first three constraints of the integer linear program as follows.

1. *N*_1, *a *_+ *N*_2, *a *_+ *N*_3_,_*a *_*+ N*_4_,_*b *_+ *N*_5,*a *_≥ ℓ - *p *- *d *+ *d*_*H*_(*x*_1_, *t*_1_).

2. *N*_1, *a *_+ *N*_2, *a *_+ *N*_3_,_*b *_+ *N*_4_,_*a *_+ *N*_5_,_*b *_≥ ℓ - *p *- *d *+ *d*_*H*_(*y*_1_, *t*_1_).

3. *N*_1, *a *_+ *N*_2, *b *_+ *N*_3,*a *_+ *N*_4,*a *_+ *N*_5,*c *_≥ℓ - *p *- *d *+ *d*_*H*_(*z*_1_, *t*_1_).

Because the integer linear program has only ten variables, checking whether it has a solution can be done in *O*(1) time. Notice that the integer linear program only depends on eight parameters *n*_1_, ... *n*_5_, *d *- *d*_*H*_(*x*_1_, *t*_1_), *d *- *d*_*H*_(*y*_1_, *t*_1_), and *d *- *d*_*H*_(*z*_1_, *t*_1_). The first five parameters are in the range [0, ..., ℓ] and the other parameters are in the range [0, ... *d*]. Therefore, we will store the results of all possible integer linear programs in a 8-dimensional table of size (ℓ + 1)^5^(*d *+ 1)^3 ^to speedup the checking time for the integer linear programs during the traversal on the tree in FullPrune. Notice that we only need to compute the table once before FULLPRUNE is executed, and reuse it as many times as needed. The pseudocode of FULLPRUNE is given below.

Algorithm FULLPRUNE

1. Compute *d*_*H*_(*x, y*) and *d*_*H*_(*x, z*).

2. Compute *n*_1_, *n*_2_, *n*_3_, *n*_4 _and *n*_5 _for each *p *= 0... (ℓ - 1).

3. Traverse the tree $T_{d}\left(x\right)$ in a depth-first manner. At each node (*t, p*), do the following steps.

(a) Incrementally compute *d*_*H*_(*x, t*), *d*_*H*_(*y, t*), and *d*_*H*_(*z, t*) from its parent.

(b) Incrementally compute *d*_*H*_(*x*_1_, *t*_1_), *d*_*H*_(*y*_1_, *t*_1_), and *d*_*H*_(*z*_1_, *t*_1_) from its parent. (Notice that *t*_1 _= *t*[1] ... *t*[*p*], *x*_1 _= *x*[1] ... *x*[*p*], *y*_1 _= *y*[1] ... *y*[*p*] and *x*_1 _= *z*[1] ... *z*[*p*]).

(c) If *d*_*H*_(*x, t*) ≤ *d, d*_*H*_(*y, t*) ≤ *d *and *d*_*H*_(*z, t*) ≤ *d*, then output *t*.

(d) Solve the integer linear program (ILP) with parameters *n*_1_, *n*_2_, *n*_3_, *n*_4_, *n*_5_, ℓ - *p *- *d *+ *d*_*H*_(*x*_1_, *t*_1_), ℓ - *p *- *d *+ *d*_*H*_(*y*_1_, *t*_1_), and ℓ - *p *- *d *+ *d*_*H*_(*z*_1_, *t*_1_).

(e) If *d*_*H*_(*x, t*) ≥ *d *and/or the ILP does not have a solution, then prune the subtree rooted at node (*t, p*). Otherwise, explore its children.

**Theorem 2.2**. *Given three *ℓ*-mers x, y and z*, FULLPRUNE*computes B*_*d*_(*x, y, z*) *in O*(ℓ + *d*|*B*_*d*_(*x, y, z*)|) *time*.

*Proof*. From the discussion above, FULLPRUNE outputs all of the ℓ-mers in *B*_*d*_(*x, y, z*). Now let us analyze its run time. In the pseudocode of FullPrune, step 1 and step 2 take *O*(ℓ) time. We will show that step 3 takes at most *O*(*d*|*B*_*d*_(*x, y, z*)|) time, that will complete our proof. Since in $T_{d}\left(x\right)$ a node and its parent differ at exactly one location, step 3a and step 3b take at most *O*(1) time. It is easy to see that the other steps inside step 3 (from step 3c to step 3e) also take *O*(1) time. Therefore, FULLPRUNE spends at most *O*(1) time at each node it visits. As a result, the run time of step 3 is proportional to the number of the visited nodes. We will argue that the number of visited nodes is no more than *d*|*B*_*d*_(*x, y, z*)|. Consider the tree T consisting of all the nodes visited by FullPrune. Obviously, $T_{d}\left(x\right)$ contains T. Because of the property of the integer linear program, every leaf in T is an element in *B*_*d*_(*x, y, z*). Therefore, the number of leaves in T is at most *B*_*d*_(*x, y, z*). On the other hand, in any tree the number of nodes is no more than its depth times the number of its leaves. Since $T_{d}\left(x\right)$ contains T, the depth of T is less than or equal to the depth of $T_{d}\left(x\right)$, which is equal to *d*. Hence, the number of nodes in T, which is equal to the number of nodes visited by FullPrune, is at most *d*|*B*_*d*_(*x, y, z*)|.

We conclude this section with a remark that our algorithm FULLPRUNE can be generalized as follows. Right now we use the computation of the common *d*-neighborhood of three ℓ-mers as the basic step. This can be generalized so that the basic step is that of computing the common *d*-neighborhood of *k *ℓ-mers (for any value of *k *≤ *n*).

### 2.3 Extended PMS5 for Solving the *q-PMS *Problem

In this section, we consider a generalized version of the PMS problem called the *q*-PMS Problem (see e.g., [22]). In the *q*-PMS problem, we relax the constraints on the motifs. An ℓ-mer *x *is a motif if there are at least *q *sequences *s*_*i *_in S such that *d*_*H*_(*x, s*_*i*_) ≤ *d*. Apparently, the *q*-PMS problem becomes the PMS problem if *q *= *n*. In practice, the *q*-PMS problem is a more realistic model of motifs since these motifs usually appear in some of the given sequences, instead of appearing in all of them.

We can extend the algorithm PMS5 to solve the *q-PMS *problem by exploiting the heart of PMS5, i.e., the algorithm FULLPRUNE that computes *B*_*d*_(*x*,*y, z*). One simple and straightforward way to extend PMS5 for the *q*-PMS problem is as follows. We consider every tuple of sequences (*s*_*i*_, *s*_*j*_, *s*_*k*_), 1 ≤ *i *<*j *<*k *≤ *n*. For each tuple (*s*_*i*_, *s*_*j*_, *s*_*k*_), we compute *B*_*d*_(*x, y, z*) where *x, y*, and *z *are in *s*_*i*_, *s*_*j *_and *s*_*k*_, respectively. For each ℓ-mer *t *in *B*_*d*_(*x, y, z*), we check whether there are at least *q*-3 sequences *s*_*p *_in $S\backslash \left\{s_{i},s_{j},s_{k}\right\}$ such that *d*_*H*_(*t, s*_*p*_) ≤ *d*. If *t *satisfies this constraint, we output *t *as a motif. The psuedocode is provided below.

**Extended Algorithm **PMS5 for *q*-PMS

1:  **for **each tuple of sequences (*s*_*i*_, *s*_*j*_, *s*_*k*_), where 1 ≤ *i *<*j *<*k *≤ *n ***do**

2:     **for **each tuple (*x, y, z*) of ℓ-mers where *x *∈_ℓ _*s*_*i*_*,y *∈_ℓ _*s*_*j*_, and *z *∈ _ℓ _*s*_*k *_**do**

3:        Compute *B*_*d*_(*x, y, z*) using FULLPRUNE.

4:        **for **each *t *∈ *B*_*d*_(*x, y, z*) **do**

5:           **if **there are at least *q*-3 sequences $s_{p}\in S\backslash \left\{s_{i},s_{j},s_{k}\right\}$ such that *d*_*H*_(*t, s*_*p*_) ≤ *d ***then**

6:             output *t*.

7:           **end if**

8:        **end for**

9:      **end for**

10:  **end for**

The two following theorems are immediate:

**Theorem 2.3**. *The worst run time of the above algorithm is *$O\left(n^{4}m^{3}dN\left(\ell ,d\right)\right)$.

**Theorem 2.4**. *The expected run time of the above algorithm is *$O\left(n^{4}m^{3}d4^{\ell }p_{\ell ,d}^{3}\right)$, *where *$p_{\ell ,d}=\sum_{i=0}^{d}\left({}_{i}^{\ell }\right){\left(3/4\right)}^{i}{\left(1/4\right)}^{\ell -i}$

### 2.4 Challenging Instances for *q*-PMS

The challenging instances for *q*-PMS have to be defined appropriately. For every value of ℓ, we can define a corresponding challenging instance with a relevant value for *d*. We define the challenging instance corresponding to a given value of ℓ to be the pair (ℓ, *d*) if *d *is the smallest value for which the expected number of (ℓ, *d*)-motifs that occur by random chance is at least 1. In fact the same definition is used for the PMS problem as well. However, the identification of such instances is slightly different. We could identify the challenging instances for *q*-PMS as follows. Let *S*_1_, *S*_2_, ..., *S*_*n *_be the given input strings. Consider a random ℓ-mer *w*. Let *S *be any one of the input strings and *x *be an ℓ-mer in *S*.

Probability that the Hamming distance between *w *and *x *is ≤ *d *is $P=\sum_{i=0}^{d}\left({}_{i}^{\ell }\right){\left(\frac{3}{4}\right)}^{i}{\left(\frac{1}{4}\right)}^{\ell -i}$. Probability that the Hamming distance between *w *and *x *is >*d *is (1 - *P*). Probability that the Hamming distance between *w *and each ℓ-mer of *S *is >*d *is *Q*' = (1 - *P*)^ℓ-*m*+1^. Here we assume that the ℓ-mers of *S *are independent, which is clearly incorrect. A similar assumption has been made in prior analyses (see e.g., [3]) and in practice conclusions made using such analyses seem to hold nearly. Probability that *S *has at least one ℓ-mer *x *such that the Hamming distance between *w *and *x *is ≤ *d *is *Q *= 1 - *Q*'. If the Hamming distance between *w *and *x *is ≤ *d*, call *x *as an instance of *w*.

Probability that *w *has at least one instance in at least *q *of the *n *input strings is $R=\sum_{i=q}^{n}\left({}_{i}^{n}\right)Q^{i}{\left(1-Q\right)}^{n-i}$. Therefore, the expected number of motifs that occur by random chance is 4^ℓ^*R*. Table 2 displays the expected number of random motifs corresponding to various values of ℓ and *d *with *n *= 20, *m *= 600 and *q *= 10. Challenging instances are shown in bold face.

**Table 2:** The expected number of random motifs for *q*-PMS corresponding to various values of ℓ and *d *with *n *= 20, *m *= 600 and *q *= 10

1	*d*	Expected Number of Random Motifs
9	2	1.599

9	1	0.159

11	2	1.424

11	1	8.643 × 10^12^

13	3	22.090

13	2	1.530 × 10^-9^

15	4	154

15	3	7.150 × 10^-8^

17	5	640

17	4	5.277 × 10^-6^

19	6	1883

19	5	8.504 × 10^-6^

Challenging instances of *q*-PMS are shown in bold face.

## 3 Results and Discussion

### 3.1 Performance of PMS5 on the challenging instances

In this section, we show the performance of PMS5 on the challenging instances as described in Section 1. We also compare the performance of PMS5 with that of other well-known exact algorithms such as Pampa [10], PMSPrune [22], Voting [20], and RISSOTO [21]. Algorithms for planted motif search are typically tested on random input datasets. Any such dataset will consist of 20 random strings each of length 600 (*n *= 20, *m *= 600). A random motif of length ℓ is planted at a random position in each of the strings, mutating it in exactly *d *places. We test the algorithms for varying ℓ and *d *values. In particular, we have employed the following challenging instances: (13, 4), (15, 5), (17, 6), (19, 7), (21, 8), and (23, 9).

To have a fair comparison, we have run all of the algorithms on the same machine. The configuration of the machine is Windows XP Operating System, Dual Core Pentium 2.4GHz CPU with 3GB RAM. PMS5 is written in C language. Pampa, PMSPrune and RISSOTO were also written in C language. Only Voting was written in C++. All of the algorithms were compiled using Microsoft Visual C++ 6.0 Compiler.

Table 3 shows the performance of the algorithms on the challenging instances. In Table 3, the letter '-' means that the corresponding algorithm either uses too much memory or takes too long on the challenging instance. In other words, the algorithm cannot solve the challenging instance in the experimental settings. We see that PMS5 outperforms the other algorithms on all of the challenging instances except on (13,4) and notably PMS5 is the only algorithm that can solve the two challenging instances (21, 8) and (23, 9). PMS5 takes more time than Pampa, PMSPrune and Voting on (13,4) because it takes an additional amount of time to load the table that stores the results of the integer linear programs. This process takes about 50 seconds. On the larger challenging instances, this amount of time is negligible.

**Table 3:** Time comparison on challenging instances

Algorithm	(13,4)	(15,5)	(17,6)	(19,7)	(21,8)	(23,9)
PMS5	117s	4.8 m	21.7 m	1.7h	9.7h	54h

Pampa	35s	6 m	40 m	4.8h	-	-

PMSPrune	45s	10.2 m	78.7 m	15.2h	-	-

Voting	104s	21.6 m	-	-	-	-

RISOTTO	772s	106.4 m	-	-	-	-

While comparing PMS5 and PMSPrune, we notice an interesting fact that as the challenging instance increases in size, the ratio between their run times increase exponentially. In particular, the ratio is roughly 2,4, and 8 on the challenging instances (15,5), (17,6), and (19,7), respectively. This fact perhaps explains why PMS5 can solve the challenging instances (21, 8) and (23, 9) but PMSPrune cannot. If this observation is true in general, PMSPrune will probably take about 16 × 9.7 = 155.2 hours on the instance (21, 8), and 32 × 54 = 1728 hours on the instance (23, 9).

Notice that the run time of PMS5 does not include the time for building the ILP table. It takes 1.5 hours and 500MB to build and store the ILP table for ℓ = 23 and *d *= 9.

### 3.2 PMS5 on real data: predicting transcript factor-binding sites

In this section, we discuss how to use algorithm PMS5 to find transcript factor-binding sites in the real data provided in [23]. The real data is broadly used to test many existing algorithms [23], [11], [22], [3]. Each dataset in the real data is comprised of DNA sequences with verified transcript factor-binding sites. The datastes are from many species including mouse, human and yeast.

We have used the algorithm PMS5 to find transcript factor-binding sites as follows. For any given dataset, we have run PMS5 with ℓ = 21, *d *= 8, and obtained a set of motifs. Some of these motifs could be spurious hits. Hence, we need a scoring scheme to rank the motifs. We have used the simple scoring function ∑_1≤*i*≤*n *_*d*_*H *_(*M, s*_*i*_), where *d*_*H*_(*M, s*_*i*_) is the hamming distance between motif *M *and sequence *s*_*i*_. We take the motif with the lowest score and then predict transcription factor-binding sites based on it. Notice that we have only used one value for ℓ (namely, 21) because smaller values of ℓ have been tested in [22].

We provide the detailed results in Table 4. In Table 4, the first column is the name of the dataset. The dataset is from mouse (resp. human) if the dataset's name starts with "mus" (resp. "hm"). The second column is the motif with the lowest score produced by algorithm PMS5. The third column is the verified transcription factor-binding sites that overlap with the predicted transcription factor-binding sites at least 60% of the motif length. We find that there are 10 out of 37 datasets in which the predicted transcription factor-binding sites are correct. In particular, one of the verified transcription factor-binding sites in dataset **hm22r **contains the predicted transcript factor-binding site. Therefore, we conclude that the results in Table 4 once again reaffirm the accuracy of the PMS model. In practice one could use PMSPrune (for values of ℓ up to 19) and PMS5 (for values of ℓ larger than 19) together to identify motifs. In this case the sensitivity will be better than using PMSPrune alone (or any of the algorithms reported in [24,25]).

**Table 4:** PMS5 on real datasets: predicting transcript factor-binding sites

Dataset	Best motif found by PMS5	**Matched binding sites at**:
mus05r	AGAGGAAAAAAAAAAGGAGAG	seq 1: GGAAAAACAAAGGTAATG

mus07r	GCTGCCCACCCTCTGCAACCC	seq 4: CCCAACACCTGCTGCCTGAGCC

mus11r	AGGGCGGGGGGCGGAGCGGGG	seq 2: GCCGCCGGGGTGGGGCTGAG
		seq 3: GGGGGGGGGGGCGGGGC
		seq 4: GTGGGGGCGGGGCCTT
		seq 9: GAACAGGAAGTGAGGCGG

hm03r	AAAAGAAAAAAAAATAAACAA	seq 1: CGGGTGTTATTCAAGCAAAAAAAATAAATAAATACCTATGCAATAC
		seq 2: GGATGTTACACAAGCAAACAAAATAAATATCTGTGCAATAT
		seq 3: TGGGTGTTATATGAGCAAACAAAATAAATACCTGTGCAACAT

hm08r	CAGCGTGCAGTCCCCTTCATC	seq 10: TATGGTCATGACGTCTGACAGAGC

hm19r	CCCCCTTCCACCACCCACAGA	seq 2: CACTTTTAGCTCCTCCCCCCA

hm20r	CCTCCTTCCTCCCCCTCCCCC	seq 10: TCCTCCCCACCTTCCCCACCCTCCCCACCCTCCCCATAAGCGCCCCTCCCG
		seq 11: GCAAACTCCGCCTCCCCCAA
		seq 14: GTCCCTCCTCCTCCCGCCC

hm22r	GGACACGGCAGAGCCTGGGGA	seq 4: GAGGCAGACCACGTGAGAGCCTGGCCAGGCCTTCC

hm24r	CGCCTGCGCCCCGCCCCGCCC	seq 2: CCCCGCCCCGCGCTCCCC

hm26r	CCCCCCGCCTCCCGCTCCCAG	seq 3: CCCCGCCTCAGGCTCCCGGGG
		seq 7: CTCAGCCTGCCCCTCCCAGGGATTAAG
		seq 8: GCGCCGAGGCGTCCCCGAGGCGC

### 3.3 Performance of Extended PMS5 on the *q*-PMS challenging instances

In this section, we show the performance of Extended PMS5 on the *q*-PMS challenging instances as described in Section 2.4. The experiment setting is the same as that in Section 3.1. Any dataset will consist of 20 random strings each of length 600 (*n *= 20, *m *= 600). We choose the parameter *q *= 10, which requires motifs to appear in at least 50% of input sequences. Note that this choice of *q *corresponds to the worst case run time (from among all possible values of *q*). Table 5 shows the run time of Extended PMS5 on the *q*-PMS challenging instances. Extended PMS5 can solve *q*-PMS challenging instances (17, 5) in 15.9 hours and it fails to solve *q*-PMS challenging instances (19, 6).

**Table 5:** Run time of Extended PMS5 on *q*-PMS challenging instances

*q*-PMS challenging instance	Run time
(9,1)	78s

(11, 2)	7.48 m

(13, 3)	18.43 m

(15, 4)	1.39h

(17, 5)	15.93h

(19, 6)	-

## 4 Conclusions

In this paper we have presented an efficient exact algorithm for the (ℓ, *d*)-motif search problem. This algorithm is more efficient than any known exact algorithm for PMS. In particular, using this algorithm we can solve the challenging instances (21, 8) and (23, 9). No prior exact algorithms could solve these instances. Therefore, we hope that PMS5 will help biologists discover longer motifs in future. Our algorithm is based on some novel ideas that will be of independent interest to solve PMS and other variations of the motif search problem. One of the basic ideas we employ is that of computing the common *d*-neighborhood of three ℓ-mers. This is done using an integer programming formulation. An open problem will be to exploit this idea to further improve the performance of our algorithm. One possible direction is to use a basic step where the *d*-neighborhood of *k *ℓ-mers is computed (for some relevant value of *k*). We have extended our algorithm to solve the *q*-PMS problem as well. Challenging instances for the *q*-PMS problem have been defined and computed. Our extended algorithm can solve the following *q*-PMS challenging instances: (9,1), (11, 2), (13, 3), (15, 4), and (17, 5). In comparison, the exact algorithms MITRA, RISOTTO, and Voting also can only solve challenging instances up to d = 5 (but for the version where the motifs occur in all the input strings).

## Authors' contributions

HD and SR designed and analyzed the algorithms. HD and VKK implemented the algorithms and carried out the empirical experiments. HD, SR and VKK analyzed the empirical results. HD and SR drafted the manuscript.

HD, SR and VKK read and approved this paper.
